# Partial Analytical Validation of microRNAs‐148a and 375 for Detecting Pancreatic Injury in Healthy Dogs and Dogs With Pancreatitis

**DOI:** 10.1111/vcp.70067

**Published:** 2025-11-18

**Authors:** Caylen Erger, Kyan Thelen Strong, Harry Cridge

**Affiliations:** ^1^ Department of Small Animal Clinical Sciences, College of Veterinary Medicine Michigan State University East Lansing Michigan USA

**Keywords:** canine, diagnosis, hemolysis, icterus, lipemia, pancreatic biomarker

## Abstract

**Background:**

microRNAs (miRNA) have been proposed as biomarkers for pancreatitis in dogs due to their high peak concentrations and experimental correlation with acinar cell injury. However, analytical validation and the effect of interfering substances are unknown.

**Objective:**

The study aimed to (i) develop and analyze the analytical validity of an assay for miRNA‐148a and miRNA‐375 in serum and (ii) compare miRNA‐148a and miRNA‐375 between healthy and pancreatitis‐affected dogs.

**Methods:**

Circulating miRNAs were quantified from serum of healthy (*n* = 40) and pancreatitis‐affected dogs (*n* = 40). Reference intervals were established, and serum miRNAs were compared between groups. Linearity was assessed via dilutional parallelism (observed/expected ratio, O/E). Precision was assessed via intra‐ and inter‐assay coefficients of variation (% CV). Intralipid, bilirubin, and hemoglobin were tested as interferants.

**Results:**

For miRNA‐148a, the RI was < 33 000 gene copies and mean O/E was 100.4%. Mean inter‐assay and intra‐assay % CV were 1.5% and 1.3% respectively. For miRNA‐375, the RI was < 4 gene copies and mean O/E was 100.1%. Mean inter‐assay and intra‐assay % CV were 3.2% and 0.5% respectively. Hemolysis led to false increases in both assays (*p =* 0.003 – < 0.0001). miRNA‐148a was reduced in dogs with pancreatitis (mean 192 gene copies) compared with healthy controls (mean 2760 gene copies; *p* < 0.0001), but miRNA‐375 was not different between groups (*p* = 0.94).

**Conclusions:**

The miRNA assays were analytically valid; however, hemolysis, a common interferant in pancreatitis cases, may impact test results. miRNA‐148a was suppressed in dogs with pancreatitis. It is unclear whether this represents a cause or a consequence of the disease.

## 1 Introduction

Pancreatitis is the most common disorder of the exocrine pancreas in dogs; however, definitive diagnosis remains challenging. Histopathology has traditionally been considered the reference standard for diagnosis of the disease, but it is uncommonly performed due to cost, its invasive nature, and the challenge associated with the multifocal distribution of inflammatory lesions in some dogs [1, 2]. Given the challenges associated with histopathology, clinicians utilize an overall assessment of clinical history, physical exam findings, ultrasonographic examination, and quantification of pancreatic lipase activity or concentration to determine whether a dog has pancreatitis [1]. Unfortunately, this diagnostic approach also has limitations related to discrepant results between ultrasound findings and lipase quantification, and an overall weak correlation with histopathologic features of disease [3, 4, 5, 6]. The lack of agreement between ultrasonographic features of pancreatitis and pancreatic lipase quantification may be due to its short half‐life (~2 h), and as such a biomarker with a longer half‐life may offer fewer diagnostic challenges in clinical practice [7]. Detection of extra‐pancreatic lipases by some catalytic assays could also contribute to this diagnostic discrepancy [8]. Pancreatic lipase can be considered a ‘leakage’ biomarker in that it enters the systemic circulation in response to pancreatic injury, most commonly pancreatitis. However, if fibrosis is present, it may limit the sensitivity of lipase‐based biomarkers, for example, in chronic pancreatitis [9, 10]. Thus, additional diagnostic tools for pancreatitis could be of clinical benefit in specific scenarios.

MicroRNAs (miRNAs) are noncoding RNAs that regulate messenger RNA expression. miRNAs have been proposed as a potential diagnostic tool in pancreatitis, as they are highly tissue‐specific, remain stable in serum, and may have a longer half‐life than traditional pancreatic lipase [11, 12]. Additionally, serum miRNA‐375 and miRNA‐148a concentrations have been shown to be closely correlated with histopathologic acinar cell injury, specifically necrosis and apoptosis, in experimental models of pancreatitis in dogs [13]. This is of particular interest as there is a lack of strong correlation between pre‐existing biomarkers and the degree of histopathologic disease in pancreatitis. Limited data currently exist regarding the utility of miRNA‐375 and miRNA‐148a in naturally occurring pancreatitis in dogs, although one study noted higher concentrations of miRNA‐375 in dogs with pancreatitis [14]. Unfortunately, data on analytical validation or the effect of interfering substances on the assay are lacking, and alternative miRNA assays remain unavailable. It is critical to investigate these factors before such an assay can be used in a clinical population.

Thus, the objectives of this study were to (i) develop and validate assays for quantification of miRNA‐148a and miRNA‐375 in canine serum and (ii) compare the quantity of these microRNAs between adult healthy dogs and pancreatitis‐affected dogs.

## 2 Materials and Methods

### 2.1 Samples

Residual serum samples from 40 healthy adult dogs (> 1 year of age) and 40 dogs with clinical signs consistent with acute‐onset pancreatitis were used in this study. Diagnosis of pancreatitis was based on consistent clinical signs (e.g., vomiting and abdominal pain), elevated Spec cPL concentration, and the attending clinician's suspicion for pancreatitis. Spec cPL has been shown to have high analytical and clinical specificity [15]. Dogs were judged to be clinically healthy based on history, physical examination by a licensed veterinarian, complete blood count, and serum biochemistry profile. Age, sex, and breed were recorded. Serum samples from pancreatitis‐affected dogs were obtained from an institutional biobank. Biobank sample collection originated from residual serum from diagnostic samples. An IACUC exemption was approved for this study due to the use of residual serum. Samples were processed and serum was separated from blood cells immediately and refrigerated for up to 12 h prior to freezing at −80°C. Samples were visually inspected for the absence of hemolysis and icterus. Serum samples were stored at −80°C in a biobank for up to 3 years prior to analysis.

### 2.2 microRNA Assay Development and Validation

Circulating microRNAs were extracted from serum using the miRNeasy Serum/Plasma Advanced Kit (Qiagen, Hilden, Germany) following the manufacturer's instructions, with 50 μL serum and 150 μL water making up the required 200 μL sample. cDNA was synthesized using the TaqMan MicroRNA Reverse Transcription Kit (Applied Biosystems, Massachusetts, USA) according to the manufacturer's instructions. Extracted RNA from a pool of healthy control patients was used to create amplified PCR products. These were then run on agarose gel to separate TaqMan assays from the DNA products, and then DNA was precipitated from gel bands. Briefly, individual gel bands were cut out using a scalpel and soaked in 1X TAE buffer. Gel bands were then ground, frozen, and reheated to create a suspension. The gel suspension was then centrifuged and reconstituted with cold ethanol and 3 M sodium acetate to precipitate DNA. A standard curve was created using these amplified and extracted PCR products, which were then serially diluted 10‐fold for a total of 7 concentrations (range of 4.33 × 10^5^ to 6.57 × 10^12^ copies per reaction) and included a non‐template control. Quantitative PCR (qPCR) was performed using the TaqMan Universal Master Mix II, no UNG (Applied Biosystems, Massachusetts, USA), with samples run in triplicate, on the QuantStudio7 Real‐Time PCR machine (Applied Biosystems, Massachusetts, USA). The following cycling conditions were used: 50°C for 2 min, 95°C for 10 min, followed by 40 cycles of 95°C for 15 s and 60°C for 60 s. Commercially available, previously characterized, ready‐to‐use TaqMan probes (Thermo‐Fisher) were used undiluted in reactions according to the manufacturer's instructions to achieve 1× final concentrations. miRNA‐16 was originally included as an endogenous reference gene; however, given the significant difference in expression between healthy controls and individuals with pancreatitis, it was determined to be unsuitable for this cohort, and data were normalized to the standard curve created with amplified PCR products and not to a housekeeping gene. TaqMan “Classic” miRNA Assay (Thermo‐Fisher, Massachusetts, USA) identification numbers are as follows: miRNA‐148a = 000470, and miRNA‐375 = 000564. Data are presented as the number of gene copies per sample. Copy number was calculated by using amplified PCR products containing only the gene of interest with the following formula where copies per μL = (DNA concentration (ng/μL) × Avogadro's number)/(length of template (bp) × conversion factor to ng × average weight of a base pair (Da)) or copies per μL = (DNA concentration (ng/μL) × [6.022 × 10^23^])/(length of template (bp) × [1 × 10^9^] × 650). CT values obtained from the linearization curve were utilized to determine intra‐ and inter‐assay coefficients of variation (precision). Intra‐assay variability was assessed using three replicates of eight samples on the same PCR plate. Interassay variability was assessed using 28 replicates of eight samples across different PCR plates. The mean, standard deviation, and coefficients of variation were calculated, where a CV < 10% was considered acceptable [16]. Coefficient of variation was calculated by running the sample in triplicate on each plate and then using the following formula: *CV =* (*Average of Ct values/Standard Deviation of Ct values*) * *100*. Linearity was assessed by dilutional parallelism using the linearization curve for each gene of interest. Serial dilutions (1:1, 1:10, 1:10^2^, 1:10^3^, 1:10^4^, 1:10^5^, 1:10^6^, 1:10^7^) were made using RNAase‐free water. Dilutional linearity was assessed by calculating the observed to expected (O/E) ratio and was compared with a target standard of 80%–120%, as previously established [16]. Best fit values and *R* ^2^ for each plate were interpolated by GraphPad using a four‐parameter logistic model with the formula: *Y* = *Bottom* + (*Top‐Bottom*)/(*1 +* (IC50/X)^HillSlope).

### 2.3 Reference Interval

Forty surplus serum samples were acquired from clinically healthy dogs as stated above and analyzed for miRNA expression. Results were used to determine the upper limit of the reference interval for the number of copies of each miRNA by calculating the upper 90th percentile. The median and range of the data were also calculated.

### 2.4 Interference Study

The effect of common interfering substances on the developed assays was evaluated, including bilirubin (icterus), hemoglobin (hemolysis), and lipid (lipemia). Bilirubin (Scripps Laboratory, San Diego, CA, USA), hemoglobin (hemolyzed red blood cells from canine blood), and lipid (Intralipid 30% IV emulsion, VWR, Radnor, PA, USA) were added at increasing concentrations (bilirubin: 7.5 mg/dL, 15 mg/dL, and 22.5 mg/dL; hemoglobin: 125 mg/dL, 250 mg/dL, 375 mg/dL, and 500 mg/dL; lipid: 300 mg/dL, 600 mg/dL, 900 mg/dL, and 1200 mg/dL) to pooled serum samples (Spec cPL: < 400 μg/L, 400–999 μg/L, 1000–2000 μg/L, and > 2000 μg/L). Finally, each sample was also tested in the absence of interfering substances (neat). Spiked and neat samples from each interferent were run on the same PCR plate.

### 2.5 Statistical Analysis

Statistical analyses were performed using commercial statistical software (Prism 10, GraphPad Software Inc., La Jolla, CA). Shapiro–Wilk testing was used to assess normality. Statistical analyses between dogs with pancreatitis and healthy controls were conducted using the nonparametric Mann–Whitney *U*‐test, and analyses of the effect of interferents were done using the nonparametric Kruskal‐Wallis test. Correlation between circulating miRNA and cPL concentrations was assessed by the nonparametric Spearman's Correlation test. Data are expressed as median and interquartile range (IQR). A *p*‐value < 0.05 was considered statistically significant. Reference intervals were established using Microsoft Excel for Windows (Microsoft) with the Reference Value Advisor v2.1 add‐in (freeware v2.1: http://www.biostat.envt.fr/reference‐value‐advisor). Reference intervals were determined from the 40 healthy dogs using a robust method without generalized Box–Cox transformation and comprised the upper 90th percentile of the fitted distribution with 90% confidence intervals calculated around the upper limit using the bootstrap method. Tukey's test was used to identify and eliminate outliers.

## 3 Results

### 3.1 Patient Demographics

This study consisted of 40 serum samples from healthy dogs and 40 banked serum samples from patients with clinical signs consistent with acute‐onset pancreatitis. Healthy controls consisted of 16 spayed females, 3 intact females, 17 neutered males, and 4 intact males. Most healthy controls were mixed breeds (*n* = 11). Other breeds represented were Golden Retriever (*n* = 6), Labrador Retriever (*n* = 5), German Shepherd (*n* = 3), Beagle (*n* = 3), French Bulldog (*n* = 3), Great Dane (*n* = 3), English Cocker Spaniel, Husky, German Wirehaired Pointer, Irish Wolfhound, Great Pyrenees, and Rottweiler. The mean age of healthy controls was 4 years (range 1–10 years).

As serum samples for dogs with suspected pancreatitis were obtained from a biobank, complete case data was not available for all dogs. Breeds represented included Australian Cattle Dog, Beagle, Husky, Shih Tzu, Miniature Pinscher, Welsh Terrier, Brussels Griffon, Cockapoo, Bichon Frise, Golden Retriever, Yorkshire Terrier, and mixed breed dogs of a mean age of 9 years (range 4–16 years). Thirty‐two serum samples were obtained from dogs with suspected acute pancreatitis. Of these, 11 had ultrasonographic evidence (pancreatic enlargement, hypoechoic pancreas, or hyperechoic peripancreatic mesentery). Three additional dogs had other known risk factors (ingestion of a high‐fat diet, concurrent phenobarbital administration). Four serum samples were from two dogs in which an acute flare of chronic disease was suspected based on historically high cPL concentrations. Four serum samples were obtained from dogs in which pancreatitis was suspected, but cPL concentrations were ultimately found to be < 400 μg/dL.

### 3.2 Micro RNA Reference Intervals, Linearity and Precision

The calculated reference interval (RI) for miRNA‐148a was < 33 000 gene copies and the RI for miRNA‐375 was < 4 gene copies. For miRNA‐148a, the mean % CV was 1.5% for intra‐assay variability and 1.3% for inter‐assay variability. The linearity study revealed O/E ratios of 95.2%–106.2% with a mean (±SD) of 100.4% (±2.1%) for miRNA‐148a. The mean inter‐assay and intra‐assay % CV for miRNA‐375 was 3.2% and 0.5% respectively, with O/E ratios of 94.5%–105.3% with a mean (±SD) of 100.1 (±2.1%).

### 3.3 Interference Study

**FIGURE 1:**
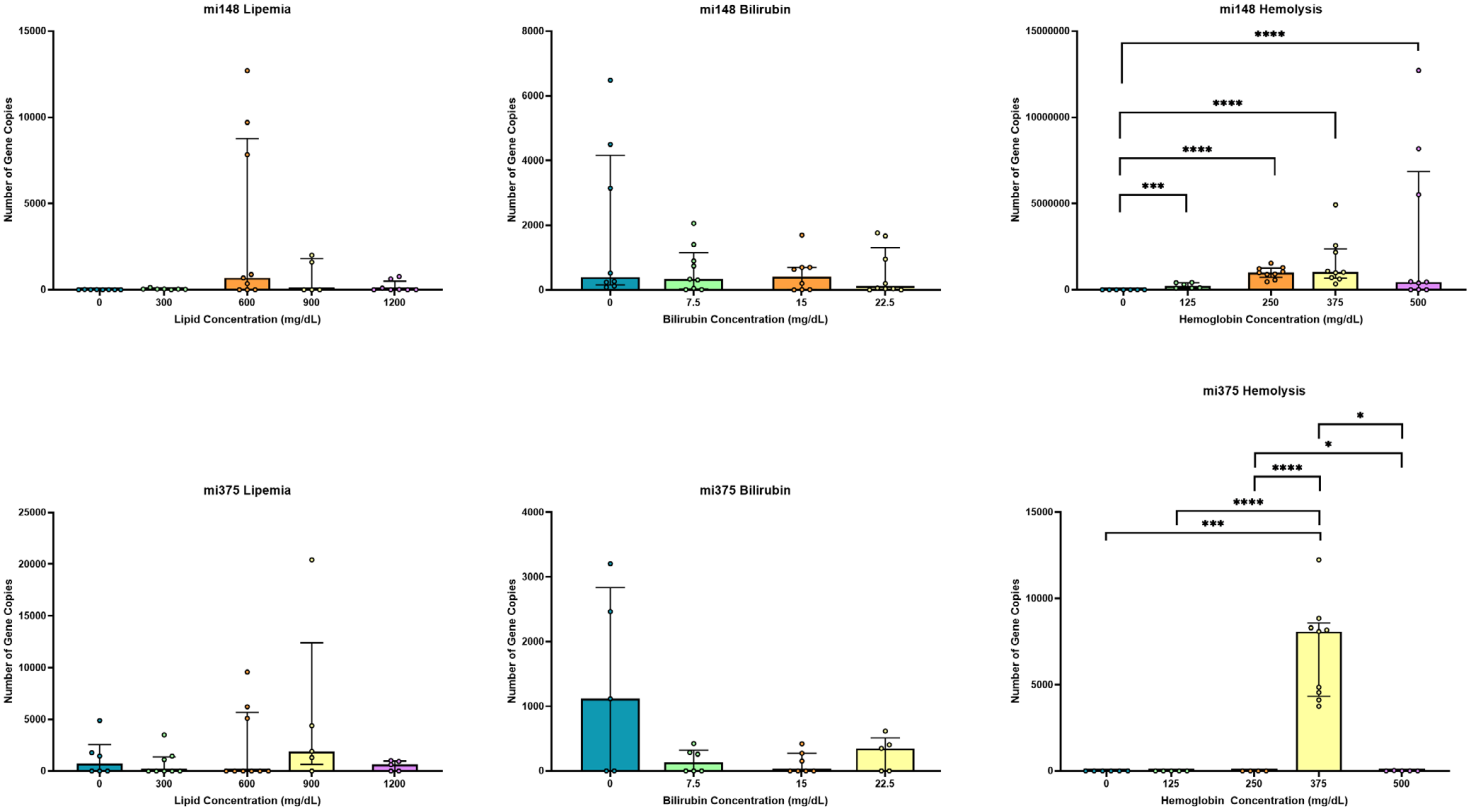
Interference study. These graphs demonstrate the effects of increasing concentrations of interferents on assay results. Hemolysis shows a statistically significant difference at all concentrations of hemoglobin, which may cause false increases in miRNA‐148a and miRNA‐375 concentrations. *, ***, **** indicates statistical comparisons.

Lipemia did not have a significant effect on gene copy number for either miRNA‐148a (*p =* 0.69) or miRNA‐375 (*p =* 0.92) (Figure 1). Similarly, icterus had no significant effect on gene copy number for miRNA‐148a (*p =* 0.23) or miRNA‐375 (*p* = 0.96) (Figure 1). Hemolysis led to a false increase in gene copy number for both miRNA‐148a (*p* < 0.0001) and miRNA‐375 (*p =* 0.003). This effect was particularly pronounced for miRNA‐148a, for which the effect was dose‐dependent across all hemoglobin concentrations (Figure 1). While miRNA‐148a and miRNA‐375 were lower in icteric samples than in neat samples, there was no significant difference detected (*p =* 0.23 and 0.96, respectively).

### 3.4 miRNA Expression in Healthy Dogs and Dogs With Pancreatitis

**FIGURE 2:**
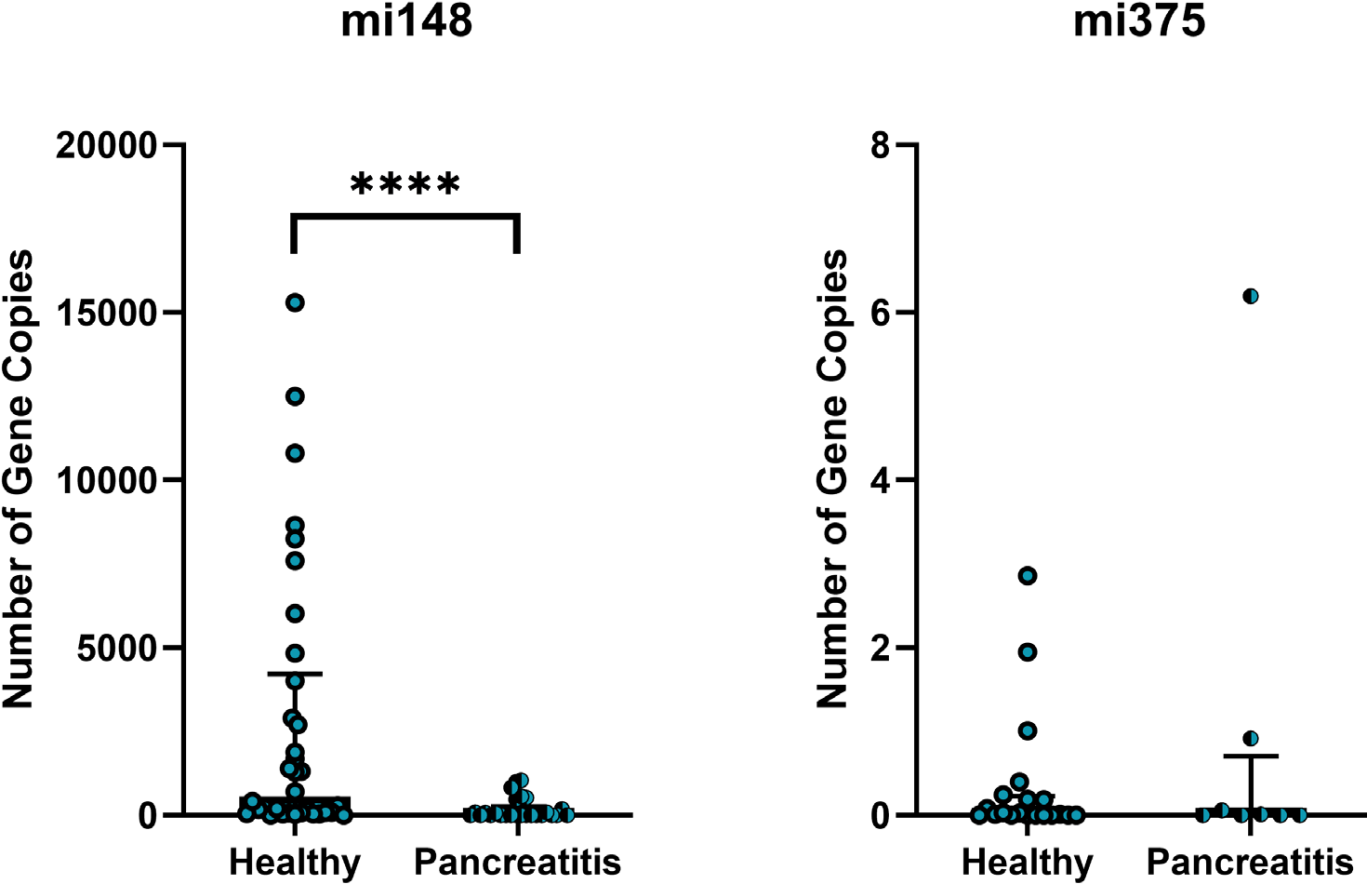
miRNA‐148a and miRNA‐375 gene copies in pancreatitis‐affected and healthy dogs. These two graphs show the difference in miRNA copies between the two study populations with outliers removed. MicroRNA 148a gene copies were significantly reduced in dogs with pancreatitis relative to healthy controls. There were no significant differences in miRNA‐375 gene copies between the two populations. **** represents a statistically significant difference between the populations.

The mean number of gene copies of miRNA‐148a (Figure 2) was significantly reduced in dogs with pancreatitis (mean 192 gene copies, range 0.24–1.53 × 10^4^, IQR 3.93 × 10^3^) compared with healthy dogs (mean 2760 gene copies, range 3.12 × 10^−14^—5.10 × 10^4^, IQR 1.57 × 10^3^; *p* < 0.0001). miRNA‐375 expression (Figure 2) was not significantly different between the two groups (*p* = 0.94). Additionally, no correlation was identified between cPL concentration and miRNA‐148a (*p* = 0.71 for healthy controls; *p* = 0.35 pancreatitis‐affected dogs) or miRNA‐375 expression (*p* = 0.62 for healthy controls; *p* = 0.50 pancreatitis‐affected dogs).

## 4 Discussion

In this study we developed assays using commercial primers for quantification of miRNA‐148a and miRNA‐375 in canine serum. We also developed reference intervals for these assays that may be utilized in future studies. The assays were found to be highly linear with an O/E ratio close to 100%, and no calculated O/E ratio was outside of the target ratio of 80%–120% as utilized in prior studies [16]. The assays were also precise with % CVs within plates and between plates close to 0%, well within the target ratio of < 10% as previously established [16]. There is variation in the miRNA copy number of the neat samples in the interference study, particularly for miRNA‐375 in the bilirubin interference group. As the same neat pools were used for all interferents, this likely indicates some degree of inter‐assay variation; however, these were not factored into the calculation of CV as the precision and interference study were performed separately and based on a pre‐determined study plan. This is a potential limitation of the precision study; however, this effect is largely driven by 3 outliers which would likely have been omitted from the precision study group had the same statistical criteria been imposed on the interference study. Therefore, it is unlikely that this observation affects the conclusion that this assay performed well analytically.

While assay analytical performance was strong, the assay was limited by the potential impacts of interfering substances. This is of particular relevance as dogs with naturally occurring pancreatitis often have hemolyzed, icteric, or lipemic serum due to their underlying disease process or challenging serum sample collection. Lipemia did not appear to affect miRNA quantification in our study, despite the use of Intralipid, which may overestimate the effects of naturally occurring lipemia [16, 17]. While icteric samples displayed a subjective false decrease in miRNA‐148a and miRNA‐375, this did not achieve statistical significance. Generally, a low isolation of miRNA‐375 may make the detection of statistically significant differences difficult. Hemolysis led to a false increase in miRNA‐148a and miRNA‐375, which would have resulted in some dogs exceeding the calculated reference interval. This may have led to a different clinical classification if the reference interval had been used in the clinical decision‐making process. While a dose‐dependent false increase was clearly noted for miRNA‐148a, this was not the case for miRNA‐375. It is possible that a more convincing dose‐dependent pattern of increase in miRNA‐375 gene copies with increasing hemoglobin concentration would emerge if higher hemoglobin concentrations were tested; however, we utilized hemoglobin concentrations based upon other studies in the field and designed to mimic clinically relevant concentrations [18]. This observation has also been noted for several other miRNA assays in dogs and may be a limitation of this diagnostic modality in dogs with pancreatitis [19]. False increases in miRNA gene copies in hemolyzed samples may result from the release of miRNAs from red blood cells, although miRNA‐148a and miRNA‐375 are not known to be enriched inside canine red blood cells [20]. The canine erythrocyte miRNAome has not been fully characterized. Future studies should consider serum quality when interpreting the clinical relevance of samples with abundant miRNA expression.

Our study sought to obtain an absolute quantification of miRNA in serum from dogs with signs consistent with pancreatitis compared with healthy dogs. Absolute quantification is lacking in the existing literature, and a validated protocol would allow for better translation between studies in different disease populations. Interestingly, our study found reduced miRNA‐148a in dogs with suspected pancreatitis compared with healthy adult dogs. MicroRNA‐148a was selected as a potential biomarker of pancreatitis in our study, as its concentration was noted to be increased in experimental models of pancreatitis concordant with the degree of acinar cell injury [13]. Differences in miRNA concentration between our study and this study may result from pre‐analytical, analytical, or patient factors. Though all samples were subjected to these same handling procedures, the duration of freezing was variable. All samples were stored at −80°C, which is not expected to lead to miRNA degradation [21]. Additionally post hoc analysis showed no effect of sample age on miRNA expression in either population. Analytical factors that could have led to the lower miRNA‐148a copy number compared with existing literature include differences in the RNA extraction, reverse transcription, or qPCR protocols. For example, cDNA was diluted following reverse transcription in preparation for qPCR, following the manufacturer's instructions. This step is necessary to minimize interference from reverse transcription reagents; however, this introduces variability in an absolute quantitative assay.

While analytical factors may account for the difference in absolute quantity compared with existing literature, the general pattern of higher miRNA‐148a expression in dogs with clinical signs of pancreatitis was expected to be conserved. As both methodologies utilized previously characterized commercial assays and identical primers, patient factors are the more likely source of discrepancy between our study and the existing literature. Patient factors that may explain the lower miRNA‐148a expression in our study include differences in patient age and size as this experimental study was conducted in juvenile dogs (< 1 year of age). However, post hoc analysis revealed no correlation between weight or age and miRNA concentration in our population of adult dogs (> 1 year of age). Other factors may include differences between experimentally induced and naturally occurring disease or differences in clinical severity or sample collection timelines.

miRNA‐148a is highly enriched in the pancreas and is derived from pancreatic islet cells [22]. miRNA‐148a has been shown to inhibit autophagy by down‐regulating the IL‐6/STAT3 signaling pathway [23]. Low miRNA‐148a expression could lead to pro‐inflammatory cytokine release thus promoting pancreatitis, although much more needs to be discovered in this area [24, 25]. Reporting of a right‐sided reference interval may seem of little value in our study as the clinically affected dogs had lower miRNA‐148a concentrations than healthy controls; however, we believe it is important to report the data here as a reference for future studies, given that our results are discordant with results in existing literature. It is plausible that our findings could prove significant for understanding the pathophysiology of pancreatic acinar cell injury based on additional future exploration.

In contrast to prior studies, miRNA‐375 was not significantly different in pancreatitis‐affected vs. healthy dogs in our study [14]. In a study of a caerulein model of pancreatic injury, serum miRNA‐375 concentrations of 10 000–10 000 copies/μL were detected up to 180 min post‐treatment with caerulein [13]. Return to baseline was demonstrated within 24–72 h in pilot experiments. Our study demonstrated far lower concentrations of miRNA‐375 in pancreatitis‐affected dogs. As some demographic and clinical data are missing in our study, discrepancies with pre‐existing data may result from patient factors, such as age, size, severity, or timeline of sample collection. However, post hoc analysis did not indicate a correlation between weight or age and miRNA concentration in our population, as previously stated. Discrepancies in miRNA‐375 results may also stem from differences in the clinical stage of dogs in each study, as miRNA‐375 has been shown to be downregulated in the acute phase of pancreatitis in rodent models [26]. The experimental study by Rouse et al. evaluated juvenile dogs of a single breed in which pancreatic injury was experimentally induced and miRNA was analyzed in the peracute period, which is not necessarily representative of a clinical population. One limitation of our study is that, while most demographic and medical data were available in our study, not all variables (e.g., ultrasound) were consistently standardized or recorded. The lack of accompanying histopathology is a limitation. The discrepant results between these studies, and the complex role of microRNAs in pancreatitis, mean that additional study is needed before microRNA assays can be utilized routinely in clinical practice.

## 5 Conclusions

The miRNA assays developed using commercial primers are analytically valid, but their interpretation may be impacted by hemolysis, a common potential interferant in clinical disease. Reduced miRNA‐148a concentrations may be seen in dogs with pancreatitis. Additional study is needed to investigate the complex dynamics of miRNAs in naturally occurring pancreatitis in dogs while remaining cognizant of sample condition interference.

## Conflicts of Interest

The authors declare no conflicts of interest.
